# Transcriptomics and overexpression analyses reveal *StERF87* confers resistance to *Ralstonia solanacearum* in potato

**DOI:** 10.1007/s00299-026-03967-7

**Published:** 2026-09-08

**Authors:** Ru Yu, Min Gao, Lin Cai, Shuoxuan Wang, Qingshuai Chen, Peiyan Guan, Caicai Lin, Xia Zhang, Shuangshuang Zheng, Lingling Jiang, Jihua Wang, Linshuang Hu

**Affiliations:** 1 Institute of Biophysics, Dezhou University, Dezhou, 253023 China; 2 Belgorod Institute of Food Sciences, Dezhou University, Dezhou, 253023 China; 3 College of Life Science, Dezhou University, Dezhou, 253023 China

**Keywords:** *Ralstonia solanacearum*, ERF transcription factors, ROS, PR protein, SA

## Abstract

**Key message:**

**StERF87 directly activates StPR1a via GCC-box binding and integrates SA/ET signaling to enhance bacterial wilt resistance.**

**Abstract:**

Potato (*Solanum tuberosum*) is an important food crop worldwide, yet its yield is severely constrained by bacterial wilt caused by *Ralstonia solanacearum*. We used RNA-Seq to study gene expression in ‘Z1076-1’ at 0, 1, and 2 days post-inoculation (dpi) with *R. solanacearum* (10⁶ CFU mL⁻^1^). We identified 6663 differentially expressed genes at 1 dpi and 7390 at 2 dpi. Calcium signaling and MAPK cascade genes were upregulated at 1 dpi. PR protein and ROS-related genes showed stronger induction at 2 dpi. The ethylene-responsive transcription factor *StERF87* was continuously upregulated. Its expression increased 4.7-fold at 2 dpi, with FPKM values over 100. We selected this gene for functional analysis. Transgenic potato plants overexpressing *StERF87* showed lower disease severity and reduced bacterial growth in both whole plants and tuber slices. StERF87 is a transcriptional activator that directly binds the GCC-box in the *StPR1a* promoter to activate its transcription. After *R. solanacearum* inoculation, *StERF87* overexpression also increased *PR1b1* expression, elevated salicylic acid and ethylene levels, reduced jasmonic acid accumulation, and altered the activities of ROS-scavenging enzymes including SOD, POD, and CAT. These results show that StERF87 regulates potato defense against *R. solanacearum* and may be useful for breeding bacterial wilt-resistant varieties.

**Supplementary Information:**

The online version contains supplementary material available at https://doi.org/10.1007/s00299-026-03967-7.

## Introduction

Potato (*Solanum tuberosum*) represents one of the most significant food crops globally and is extensively cultivated as a staple food for over 1.3 billion people (Stokstad 2019). Nevertheless, both the productivity and quality of this species are severely compromised by a diverse array of pathogenic infections. Among these, bacterial wilt triggered by *Ralstonia solanacearum* is exceptionally destructive; it has affected potato production across approximately 80 countries and inflicted annual economic losses exceeding $950 million (Patil et al. 2012). Under favorable conditions, *R. solanacearum* penetrates the host root system through wounds, root tips, or lateral root emergence sites, subsequently colonizing xylem vessels and proliferating extensively, eventually resulting in plant wilting and mortality (Lowe-Power et al. 2018; Xue et al. 2020). Despite considerable research, management strategies that combine efficacy with environmental sustainability remain insufficient for this pathogen (Jiang et al. 2017). Therefore, deciphering the molecular underpinnings of potato resistance against *R. solanacearum* is fundamental to establishing efficacious intervention measures, among which the breeding of resistant varieties is of paramount importance (Huet 2014).

Possession of the AP2 domain, a conserved DNA-binding segment consisting of roughly 60–70 amino acids, characterizes the APETALA2/ethylene response factor (AP2/ERF) transcription factor family (Owji et al. 2017). This superfamily can be divided into five separate clades, namely AP2, ERF, DREB, RAV, and Soloist, according to AP2 domain counts and other structural traits (Sakuma et al. 2002). Of particular interest, ERF clade members exert essential roles in phytohormone signal transduction, biotic‑ and abiotic-triggered stress adaptation, plant developmental events, as well as the modulation of metabolic pathways (Su et al. 2025). By physically binding to the GCC-box cis-acting element, ERF subgroup proteins regulate ethylene-related signaling cascades and improve plant performance under abiotic stress (Hao et al. 1998).

A broad spectrum of plant species remains vulnerable to bacterial infection, with disease manifestation encompassing foliar lesions, tissue blight, systemic wilting, and stem cankers. In rice, constitutive overexpression of *OsBIERF3* has been demonstrated to simultaneously strengthen defensive capacity against both fungal and bacterial invaders (Hong et al. 2022). Moreover, in wild-type Chinese grapevines (*Vitis quinquangularis*), three ERF members—*VqERF112*, *VqERF114*, and *VqERF072*—act as positive regulators of defense against *Pseudomonas syringae* pv. *tomato* DC3000 and *Botrytis cinerea* (Wang et al. 2020b). By contrast, ERF121 in *Brassica oleracea* promotes susceptibility to *Xanthomonas campestris* pv. *campestris*, as conserved TAL effectors target and activate its expression to compromise host immunity (Zlobin et al. 2021).

Previous studies have demonstrated that ERF transcription factors predominantly modulate biological stress responses via plant hormone signaling pathways, including ethylene (ET), jasmonic acid (JA), and salicylic acid (SA) (Amorim et al. 2017; Phukan et al. 2017). In apple, MdERF11 enhances resistance to *Botryosphaeria dothidea* by promoting the expression of genes related to SA synthesis (Wang et al. 2020a). *Stemphylium lycopersici* infection and exogenous SA treatment strongly induced the expression of *SlERF01*, thereby enhancing tomato resistance to this pathogen (Yang et al. 2020). *VaERF16*, as a positive regulator, enhances resistance of *Vitis amurensis* to *Botrytis cinerea* by potentiating ET/JA-dependent hormone responses (Zhu et al. 2022). In hot pepper, overexpression of *CaERF1A* upregulates genes related to ET/JA synthesis, thereby enhancing resistance to the necrotrophic fungus *Alternaria alternata* (Huh 2022). By contrast, RNAi-mediated silencing of *SlPti5* in tomato attenuates ET/JA signaling and compromises resistance to *B. cinerea* (Tang et al. 2022). Conversely, exogenous application of ET, JA, and SA induces *OsERF83* expression, and its overexpression in transgenic rice activates pathogenesis-related genes and markedly strengthens resistance against rice blast caused by *Magnaporthe oryzae* (Tezuka et al. 2019).

Accumulating evidence indicates that members of the ERF family contribute significantly to plant immunity through modulation of lignin production. In poplar, ERF139 coordinates xylem cell expansion and secondary cell wall deposition by promoting guaiacyl-type lignin and xylan accumulation, thereby enhancing tolerance to biotic stress (Wessels et al. 2019). In Arabidopsis, AtERF114 facilitates lignin deposition and boosts defense responses by directly binding to the GCC-box in the *AtPAL1* promoter, thereby activating transcription of this lignin biosynthetic gene (Li et al. 2022). Similarly, MdERF114 elevates lignin levels through direct activation of *MdPRX63* expression, resulting in enhanced lignin deposition and improved resistance to *Fusarium solani* in apple (Liu et al. 2023). However, the MaERF110-MaMYB308 transcription module has a negative regulatory effect on banana lignin-mediated Fusarium blight defense (Li et al. 2026). These findings illustrate the dual roles of ERF family members in either promoting or suppressing lignin-mediated defense, depending on the specific protein-target interaction.

This investigation employed transcriptome sequencing (RNA-Seq) to elucidate the defense mechanisms activated in potato plants following infection by *R. solanacearum*. As a robust methodology for transcriptomic analysis, RNA-Seq enables comprehensive profiling of genes exhibiting altered expression patterns across diverse physiological conditions. Our findings revealed that differentially expressed genes were predominantly associated with pathways governing plant-pathogen recognition, secondary metabolite production, and amino acid metabolic processes. Among them, the expression of four ERF genes was significantly different, and *StERF87* was one of them, which was identified as a typical ERF subfamily protein containing only one AP2 domain (Charfeddine et al. 2015). Functional analyses indicate that the overexpression of *StERF87* can enhance potato resistance to bacterial wilt by directly activating *PR1a* and indirectly regulating the activity of ROS-related enzymes, as well as the levels of SA, JA, and ET, thereby promoting the immune response in potatoes. Collectively, these results indicate that *StERF87* positively regulates potato resistance to *R. solanacearum*. The successful generation of a bacterial wilt-resistant transgenic potato line in this study holds promise for molecular breeding of disease-resistant crops.

## Materials and methods

### Plant materials and growth conditions

The potato variety ‘Z1076-1’ was used in this study and was obtained from Hissen Potato Industry Group Co., Ltd. (Dezhou, China). Plant materials were raised under greenhouse conditions: temperature set to 26 °C, photoperiod of 16 h light followed by 8 h dark, and relative humidity kept between 60% and 70%.

### *Ralstonia solanacearum* inoculation

*R. solanacearum* stocks preserved at −80 °C were streaked onto TTC solid medium and cultured for 48 h at 28 °C. Individual colonies were picked and sub‑cultured on new TTC medium for strain activation, followed by resuspension in 10 mM MgCl₂ solution to achieve a cell density of 10⁶ CFU mL⁻^1^. Potato seedlings with five to six expanded leaves received root-drench inoculation using the prepared bacterial suspension. For mock controls, plants were supplied solely with 10 mM MgCl₂ solution. Disease symptoms were scored on a daily basis with a 0–5 grading scale modified from Winstead and Kelman (1952): 0 = no visible wilting symptoms; 1 = wilting observed on one leaf; 2 = wilting of two to three leaves; 3 = wilting across most leaves, with only the top two or three leaves remaining healthy; 4 = complete wilting of all leaves; 5 = plant mortality. The Disease Severity Index (DSI), expressed as a percentage, was computed via the formula: DSI (%) = [Σ(nᵢ × vᵢ) / (V × N)] × 100. In this equation, nᵢ represents the count of plants assigned disease grade vᵢ, V corresponds to the highest disease score (5), and N indicates the total number of assessed plants.

### RNA isolation and transcriptome profiling

Clean reads were aligned against the *S. tuberosum* reference genome (DM v6.1). Transcript assembly and quantification were performed by StringTie software based on genome-mapping outputs (Pertea et al. 2015). This program adopts a network‑flow algorithm for transcript and gene assembly as well as abundance calculation. The edgeR package was applied to detect statistically significant expression variations. Statistical significance was evaluated via p‑value or padj (adjusted significance level, namely q‑value). Specifically, padj corresponds to p‑values corrected by the BH algorithm to mitigate high false‑positive rates. Lower adjusted p‑values indicate stronger statistical significance, and padj < 0.05 was set as the threshold for differential expression (Robinson et al. 2010). Hierarchical clustering, a widely‑adopted approach, was conducted on log₁₀(FPKM+1) transformed values, and clustering outputs were visualized in heatmaps. Genes satisfying |log₂FC|≥ 1 together with padj < 0.05 were regarded as differentially expressed genes (DEGs). GO (Gene Ontology) and KEGG (Kyoto Encyclopedia of Genes and Genomes) enrichment analyses for DEGs were implemented with clusterProfiler, where padj < 0.05 was defined as the cutoff for significant enrichment.

### Quantitative real-time PCR (qRT-PCR) analysis

Total RNA was reverse-transcribed into cDNA with the Evo M-MLV Plus 1st Strand cDNA Synthetic Kit (Accurate Biology, Hunan, China). qRT-PCR was run on a BioRad CFX96 real-time thermal cycler. Target transcript expression was normalized to the housekeeping gene *Stef1-α* (GenBank: AB061263.1). Three biological and three technical replicates were set for each sample reaction. Primer sequences are provided in Supplementary Table S5.

### Molecular cloning of *StERF87* and construction of the expression vector

The full-length CDS of *StERF87* (Soltu.DM.05G020900) was retrieved from Spud DB (https://spuddb.uga.edu/). Gene-specific primers carrying XbaI and SacI restriction sites were used to amplify this CDS from potato cDNA with Invitrogen Platinum SuperFi II DNA polymerase (Thermo Fisher Scientific, Waltham, MA, USA). After fragment purification, the product was inserted into the pBI121 vector by T4 DNA ligase (Takara Bio, Kusatsu, Japan) to generate the recombinant plasmid pBI121-FLAG-*StERF87* (35S:*StERF87*). The construct was transformed into *Escherichia coli* DH5α competent cells (Vazyme, Nanjing, China). Verified positive clones were cultured in 10 mL LB liquid medium containing 50 μg mL⁻^1^ kanamycin at 37 °C with shaking for 12 h. Bacterial pellets were harvested by centrifugation (12,000×g, 15 min), and endotoxin‑free high‑purity plasmid DNA was isolated with the FastPure EndoFree Plasmid Maxi Kit (Vazyme, Nanjing, China). The resulting purified plasmid was then transformed into *Agrobacterium tumefaciens* EHA105 competent cells (Vazyme, Nanjing, China) for subsequent potato genetic transformation.

### Potato transformation

For plant transformation, potato explants exhibiting approximately five lateral shoots were selected. Stem segments were excised from these donor plants and subjected to dedifferentiation culture for 3 days. Subsequently, the resulting callus tissues were immersed in *Agrobacterium* suspensions (OD₆₀₀ = 0.5) harboring the 35S:*StERF87* construct for 20 min. Stem segments were blotted dry with filter paper before being placed onto MS medium supplemented with 0.2 mg L⁻^1^ naphthalene acetic acid (NAA), 0.02 mg L⁻^1^ gibberellin (GA₃), and 2.5 mg L⁻^1^ zeatin (ZR), followed by a 2-day dark incubation. These explants were rinsed two to three times using sterile water with 300 mg L⁻^1^ timentin, and then washed another 2–3 times in MS solution supplemented with the same concentration of timentin. Upon air-drying, the explants were cultured on MS medium added with 300 mg L⁻^1^ timentin for 10 days. Subsequently, materials were shifted to selective bud-induction medium, which consisted of MS basal medium plus 0.02 mg L⁻^1^ NAA, 0.02 mg L⁻^1^ GA₃, 2 mg L⁻^1^ ZR, 300 mg L⁻^1^ timentin and 50 µg mL⁻^1^ kanamycin. Regenerated adventitious shoots were dissected and moved to rooting medium (MS containing 300 mg L⁻^1^ timentin and 50 µg mL⁻^1^ kanamycin) for root formation. The stepwise in vitro regeneration process, from initial explant culture to whole plantlet formation, is illustrated in Supplementary Figure S1. Rooted plantlets were propagated.

### PCR analysis

Total RNA was extracted from Z1076-1 and transgenic potato lines with the RNAprep Pure Plant Kit (Tiangen Biochemical Technology Co., Ltd., Beijing, China) for *StERF87* expression validation. Purified RNA was reverse-transcribed to generate first-strand cDNA using the Evo M-MLV Plus first-chain cDNA synthesis kit (Accurate Biology, Hunan, China). Amplified PCR fragments were separated by 1% agarose gel electrophoresis and DNA bands were detected under UV light.

### Western blot analysis

Total protein was extracted from ~100 mg fresh leaf tissues of wild-type potato Z1076-1 and transgenic lines OE#1, OE#2. The samples were snap-frozen in liquid nitrogen and ground into fine powder. The powder was homogenized using Plant RIPA Lysis Buffer (Beyotime Biotech Inc., Shanghai, China). Equal amounts of protein were separated by SDS-PAGE and electroblotted onto PVDF membranes for immunoblot analysis. Membranes were probed with anti-FLAG or anti-Actin antibodies (1:5000 dilution). Horseradish peroxidase-conjugated goat anti-rabbit IgG (Abcam, Cambridge, UK) served as the secondary antibody.

### Tuber slice inoculation assay

Potato tubers received surface sterilization. Soaked in 3% (v/v) NaOCl solution for 1 min, they were rinsed three times with sterile double‑distilled water and cut into ~1 cm‑thick slices. Each slice was immersed in bacterial suspension adjusted to 10⁶ CFU mL⁻^1^ for 30 min, and excess liquid was blotted off afterward. All processed slices were incubated at 28 °C in a sterile humid chamber, while tuber slices for negative‑control treatments were placed in sterile 10 mM MgCl₂ solution under identical experimental parameters.

### Bacterial population assay

Approximately 0.5 g tuber tissue slices received surface sterilization in 75% ethanol over 30 s, then washed twice using sterile water. A pre-sterilized mortar and pestle was applied to homogenize these processed samples within 1 mL sterile 10 mM MgCl₂ solution. The resulting homogenate was serially diluted with sterile MgCl₂ buffer to obtain gradients from 10⁻^1^ up to 10⁻^4^. For each diluted fraction, 100 μL aliquots were plated on TTC solid medium. The plates were incubated at 28 °C for 48 h, and bacterial colonies were counted after this cultivation period.

### Measurement of physiological indicators

Fresh potato leaf tissues (0.1 g) were ground in liquid nitrogen and homogenized in 2 mL 0.1 M phosphate buffer (pH 7.0) supplemented with 1 mM EDTA-Na₂, 1% (w/v) PVP, and 0.1% (v/v) Triton X-100. After vigorous vortexing, the homogenate was incubated on ice for 10 min. Cell debris was removed by centrifugation at 13, 000 × g for 10 min at 4 °C, and the resulting supernatant was collected as crude enzyme extracts for subsequent biochemical analyses. Reactive oxygen species (ROS) were quantified using a commercial detection kit (Nanjing Senbeijia Biological Technology Co., Ltd., Nanjing, China) according to the manufacturer’s instructions. Superoxide dismutase (SOD), peroxidase (POD), and catalase (CAT) activities were assayed with corresponding kits from Solarbio Science & Technology Co., Ltd. (Beijing, China), following the kit‑provided protocols.

### Transactivation activity assay in yeast

The complete open reading frame (ORF) of StERF87 was inserted into the pGBKT7 vector to produce the GAL4 BD recombinant fusion plasmid pGBKT7-StERF87. The resulting recombinant construct, together with the negative control plasmid pGBKT7-Lam and positive control plasmid pGBKT7-p53, was introduced into Y2HGold yeast competent cells. Positive transformants were first screened on SD/-Trp (DDO) solid medium and subsequently inoculated onto SD/-Trp/-His/-Ade/X-α-Gal (QDO) plates. Following incubation at 30 °C for 3 to 5 days, the self-activation ability of the target protein was assessed based on the growth status and blue pigmentation of yeast colonies.

### Determination of phytohormone content

Gas chromatography was applied to determine ET biosynthesis rates in accordance with the previously established protocol reported by Spollen et al. (2000). The quantification of JA and SA was performed with an HPLC–MS/MS system, and sample pretreatment of 100 mg specimens followed the experimental method documented by Baccio et al. (2012). All experimental assays were conducted with no fewer than three biological replicates for analytical validation.

### Chromatin immunoprecipitation‐qPCR

Leaf blades from tissue-cultured seedlings at 1 month of age were treated with 1% (w/v) formaldehyde solution for cross-linking. Subsequently, chromatin separation and ultrasonic fragmentation were performed, followed by immunoprecipitation using FLAG antibody. The resulting antibody-chromatin complex was precipitated using protein A/G agarose beads. After thorough washing, reverse cross-linking and protein digestion treatments were carried out. Finally, the purified DNA was used for qPCR analysis; the primers used are listed in Supplementary Table S5.

### Dual‐Luciferase reporter assays

To verify that StERF87 exerts direct regulation on the promoters of *StPR1a* and *StPR1b1*, transient luciferase reporter assays were carried out in *Nicotiana benthamiana*, using the Dual‑Luciferase^®^ Reporter Assay System (Promega). Promoter fragments of *StPR1a* and *StPR1b1* were amplified from potato genomic DNA; their corresponding primer sequences are available in Table S5. These amplified fragments were inserted into the pGreenII 0800-LUC vector. This manipulation yielded proStPR1a::LUC and proStPR1b1::LUC reporter constructs to drive LUC expression. Overlap-extension PCR was used to alter the core AGCCGCC motif within the *StPR1a* promoter. This key sequence was substituted with AAAAAAA to produce the mutated mproStPR1a::LUC construct. The full-length coding sequence of *StERF87* was cloned into pGreenII 62-SK, generating the effector plasmid 35S::*StERF87*. A REN-expressing vector was coinfiltrated alongside other plasmids. It served as an internal control for data normalization and relative promoter activity was calculated as the LUC/REN ratio.

### Statistical analysis

All experiments included at least three independent biological replicates. The raw measurements are presented as mean ± standard deviation (SD). GraphPad Prism 10.1.2 (GraphPad Software, San Diego, CA, USA) handled all statistical computations in this work. The two‑tailed Student’s t‑test served to assess disparities between transgenic materials and wild‑type (WT) controls; asterisks mark statistical significance (*P < 0.05, **P < 0.01, ***P < 0.001). For pairwise contrasts across diverse genotypes collected at the same time point, Tukey’s multiple‑comparison test was applied. Distinct lowercase letters denote meaningful differences when *P* < 0.05.

## Results

### Phenotype and pathogen colonization following bacterial infection

**Fig. 1:**
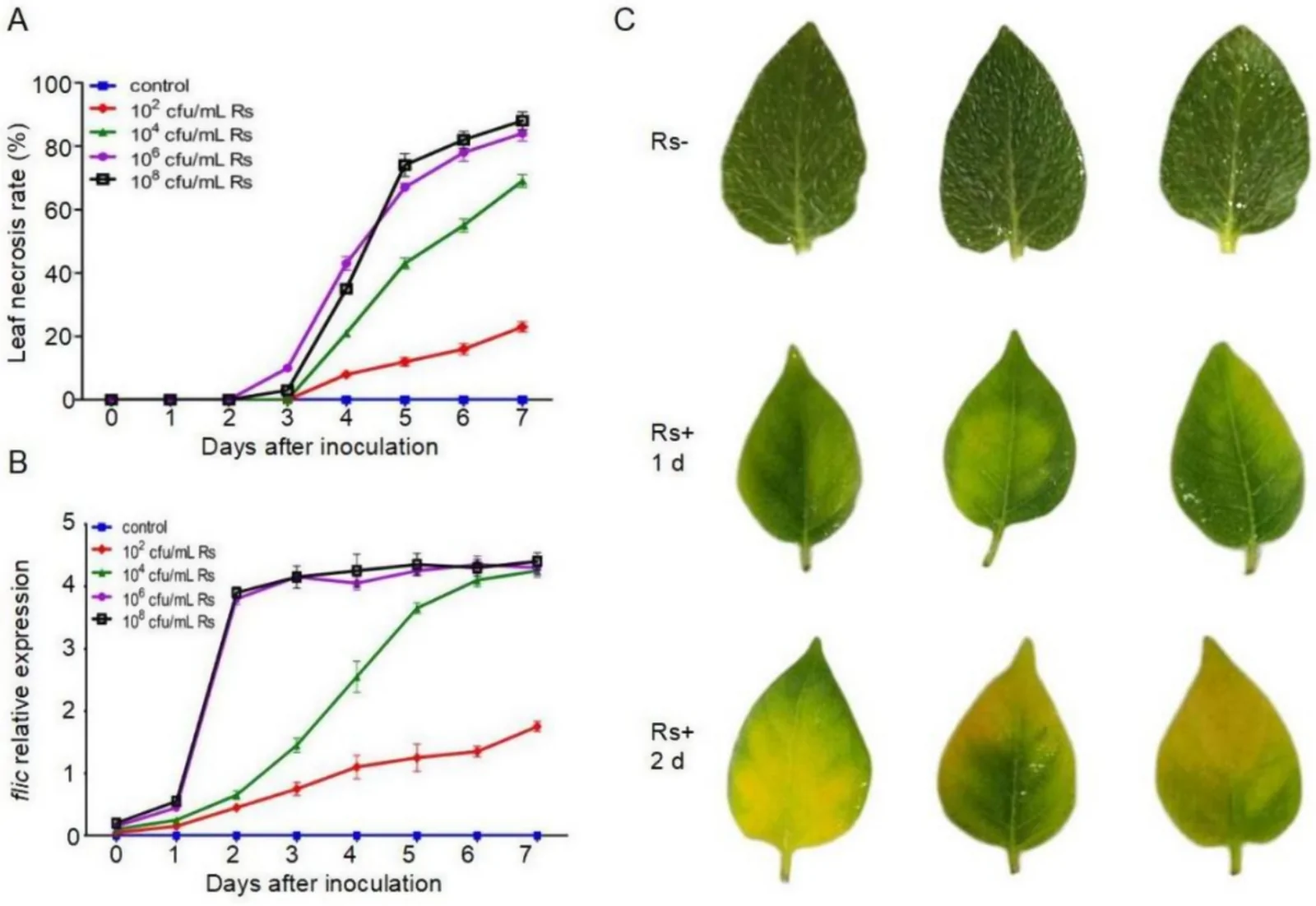
Pathological and molecular characterization of potato bacterial wilt under different inoculum concentrations and time points. **A** Leaf necrosis rates of potato plants inoculated with *Ralstonia solanacearum* at 10^2^, 10^4^, 10^6^, and 10^8^ CFU mL⁻^1^ over a 7 day period. **B** Relative expression of the pathogen-specific marker gene *fliC* in potato leaves following inoculation with *R. solanacearum* at different concentrations. **C** Representative leaf phenotypes of mock-inoculated control (Rs⁻; 10 mM MgCl_2_) and *R. solanacearum*-inoculated (Rs⁺) plants at 1 and 2 dpi. Data in A and B are presented as means ± SD from three biological replicates

Compared with mock-inoculated controls, potato plants inoculated with *R. solanacearum* exhibited progressive wilting and yellowing of leaves, accompanied by stem necrosis. Disease severity was evaluated based on the extent of leaf necrosis and pathogen colonization levels. As shown in Fig. 1A, the rate of leaf necrosis increased with rising *R. solanacearum* inoculum concentration. At concentrations of 10^6^ and 10^8^ CFU mL⁻^1^, obvious necrosis was observed from 3 days post-inoculation (dpi), whereas at 10^2^ and 10^4^ CFU mL⁻^1^, necrosis became evident at 4 dpi.

To monitor pathogen proliferation, the expression of *fliC* (a marker gene of *R. solanacearum*) was quantified by qRT-PCR. The results showed that *fliC* expression was significantly elevated at 2 dpi in plants inoculated with 10⁶ and 10⁸ CFU mL⁻^1^ (Fig. 1B), indicating rapid pathogen multiplication. Based on these findings, an inoculum concentration of 10⁶ CFU mL⁻^1^ was selected for subsequent transcriptome analysis, with samples collected at 1 and 2 dpi. At these time points, the pathogen population had reached high levels while visible necrotic symptoms had not yet developed (Fig. 1C), enabling capture of early defense responses prior to extensive tissue damage.

### Transcriptome dynamics upon *R. solanacearum* infection

To identify metabolic pathways and differentially expressed genes (DEGs) involved in potato defense against *R. solanacearum*, transcriptome profiling was performed using samples collected before inoculation (0 dpi) and at 1 and 2 days post-inoculation (1 and 2 dpi). Each treatment comprised three independent biological replicates. High-throughput sequencing generated over 79 million clean reads per library, with Q20 and Q30 quality scores exceeding 97.41% and 92.78%, respectively. Additionally, 71.49% of clean reads were mapped to the *Solanum tuberosum* reference genome across all nine libraries, indicating high data quality suitable for downstream analyses (Table S1).

Gene expression levels were quantified as fragments per kilobase of transcript per million mapped reads (FPKM) to enable cross-sample comparison (Fig. S2A). The expression distributions were relatively consistent across all samples, confirming data stability. Hierarchical clustering of log_10_(FPKM + 1)-transformed values revealed that control samples (Stc) and infected samples (St1Rs and St2Rs) segregated into distinct branches (Fig. S2B), indicating that *R. solanacearum* infection triggered substantial transcriptional reprogramming. Genes with similar expression patterns were clustered together, suggesting potential co-regulation within shared metabolic or signaling pathways.

**Fig. 2:**
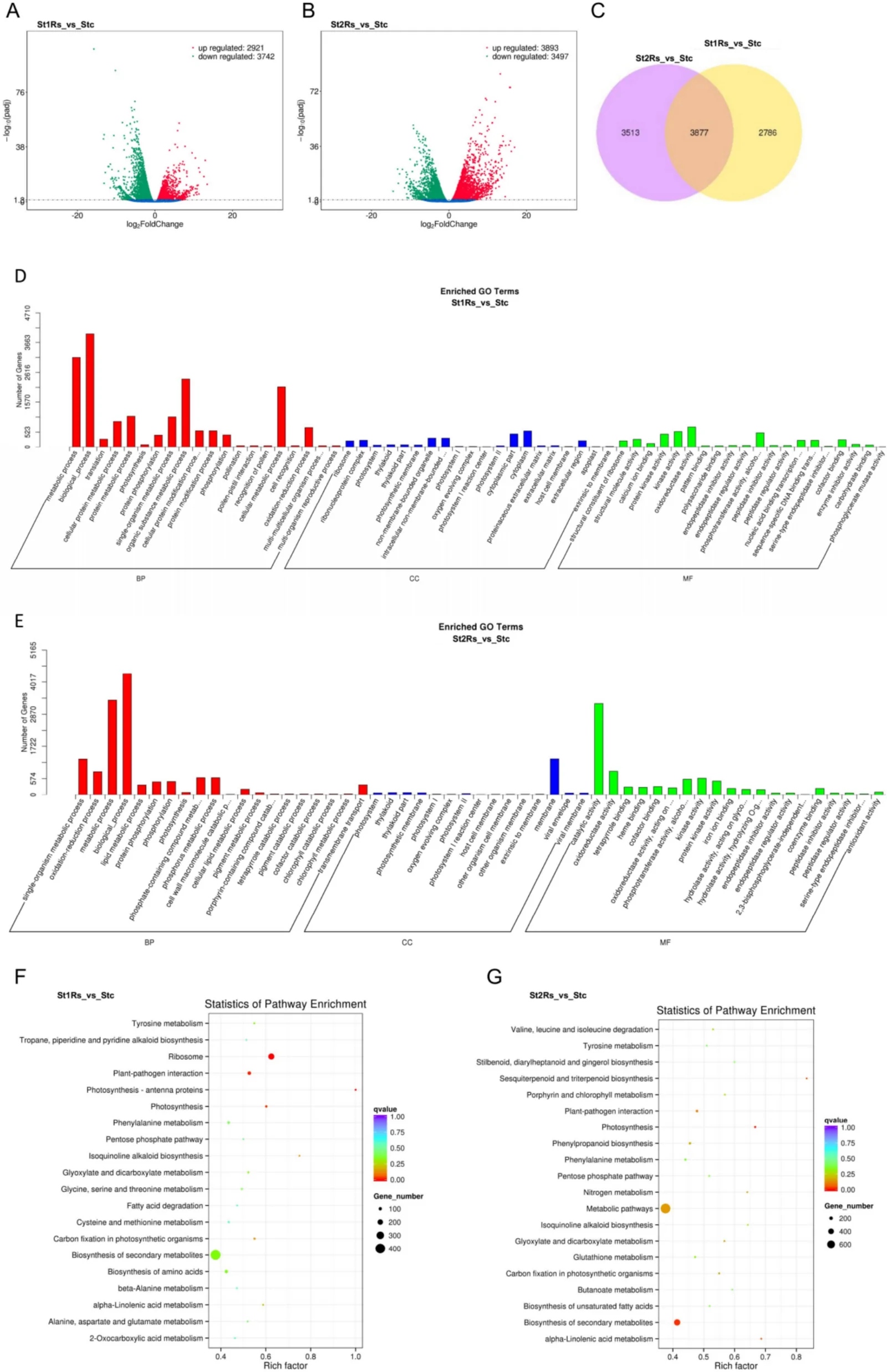
Identification and functional enrichment analysis of differentially expressed genes (DEGs) in potato following *R. solanacearum* inoculation. **A** Volcano plot of DEGs between St1Rs and Stc. **B** Volcano plot of DEGs between St2Rs and Stc. Red and green dots indicate significantly upregulated and downregulated genes, respectively (padj < 0.05 and |log2FoldChange|≥ 1). The horizontal dashed line indicates the significance threshold (padj = 0.05). **C** Venn diagram showing the overlap of DEGs between St1Rs_vs_Stc and St2Rs_vs_Stc comparisons. **D–E** Gene Ontology (GO) enrichment of DEGs shared by Stc_vs_St1Rs (**D**) and Stc_vs_St2Rs (**E**). GO terms are grouped into three categories: biological process (BP), cellular component (CC), and molecular function (MF), distinguished by red, blue, and green bars, respectively. The y-axis indicates the number of enriched genes for each GO term. F–G, Kyoto Encyclopedia of Genes and Genomes (KEGG) pathway enrichment of DEGs shared by Stc_vs_St1Rs (**F**) and Stc_vs_St2Rs (**G**). The x-axis represents the rich factor (proportion of DEGs relative to all genes in the pathway), the y-axis lists enriched pathways, circle size indicates the number of enriched genes, and color intensity (red gradient) represents statistical significance. St1Rs, St2Rs, and Stc denote biological replicates collected at 1 dpi, 2 dpi, and 0 dpi (control), respectively

Volcano plot analysis revealed that comparison of Stc with St1Rs identified 6663 differentially expressed genes (DEGs), of which 2921 were upregulated and 3742 were downregulated. Similarly, comparison of Stc with St2Rs identified 7390 DEGs, including 3893 upregulated and 3497 downregulated genes (Fig. 2A, B). Venn diagram analysis showed that 3877 genes were differentially expressed in both comparisons (Fig. 2C). To investigate the biological functions of these DEGs, Gene Ontology (GO) and Kyoto Encyclopedia of Genes and Genomes (KEGG) enrichment analyses were performed (Fig. 2D–G).

**Fig. 3:**
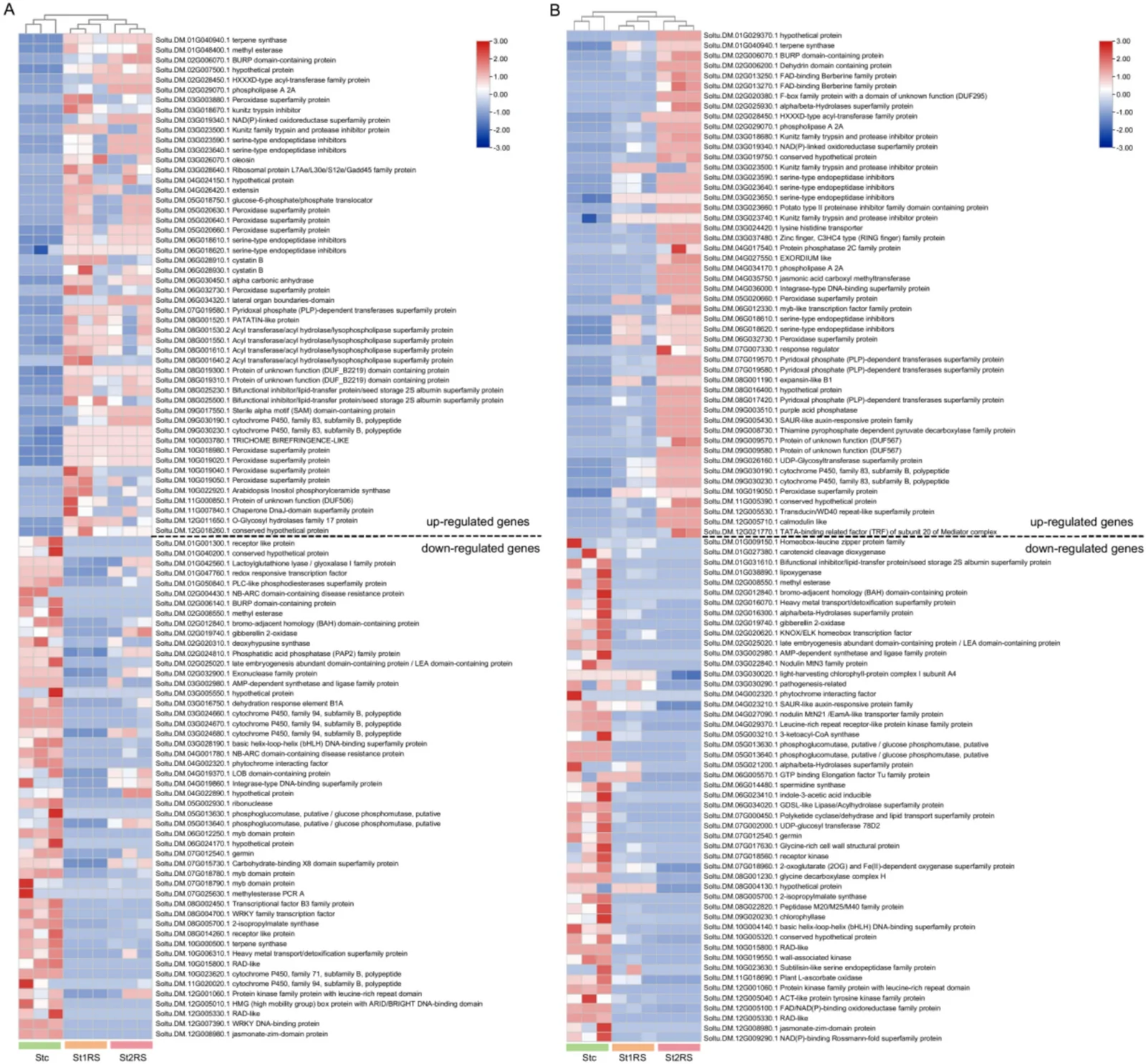
Heatmaps of differentially expressed genes (DEGs) in potato after inoculation with *R. solanacearum*. **A** Top 50 upregulated and top 50 downregulated genes identified at 1 dpi. **B** Top 50 upregulated and top 50 downregulated genes identified at 2 dpi. Expression values are shown for all sampling time points: 0 dpi (control, Stc), 1 dpi (St1Rs), and 2 dpi (St2Rs). The color scale from blue to red indicates low to high expression

GO enrichment analysis revealed that the main enriched molecular function terms included catalytic activity, oxidoreductase activity, and protein phosphorylation; the primary biological process terms comprised metabolic process and lipid metabolic process (Fig. 2D, E). KEGG pathway analysis further indicated that these DEGs were significantly enriched in pathways associated with plant–pathogen interactions, biosynthesis of secondary metabolites, and amino acid metabolism (Fig. 2F, G). These results suggest that *R. solanacearum* infection significantly modulates redox homeostasis, protein phosphorylation, and plant–pathogen interaction pathways in potato.

After *R. solanacearum* inoculation, potato exhibited significant transcriptional reprogramming at both 1 and 2 dpi (Table S3 and S4). The first 50 upregulated genes and the first 50 downregulated genes were selected for clustering in a heat map (Fig. 3). Upregulated genes were mainly enriched in categories related to defense mechanisms, including peroxidase superfamily proteins, cytochrome P450s, WRKY transcription factors, disease-resistant proteins containing the NB-ARC domain, and protease inhibitors such as Kunitz trypsin inhibitors and cystine B. In contrast, downregulated genes were primarily associated with primary metabolism processes, such as phosphoglucomutase, germin proteins, and carbohydrate-binding proteins. It is worth noting that compared to the 1^st^ day after inoculation, the expression patterns of jasmonic acid signaling-related proteins and those containing the LEA domain changed on the 2^nd^ day after inoculation. This indicates that during the process of resistance to *R. solanacearum*, the immune response in potatoes undergoes a sequential transition from an early, basic immune response to a later, hormone-mediated specific defense mechanism.

### Temporal dynamics of defense-related DEGs

Within the plant–pathogen interaction pathway (ko04626), 85 DEGs were identified in cultivar ‘Z1076-1’ that are implicated in pathogen recognition and defense signaling (Table S2). These genes were functionally categorized into several regulatory modules. At 1 dpi, genes associated with calcium signaling, ethylene response, and oxidative burst were prominently upregulated, including *CML1* (calmodulin-like protein), *CDPK17* and *CDPK25* (calcium-dependent protein kinases), *CNGC2* (cyclic nucleotide-gated channel), *ERF80*, *ERF87*, *ERF98*, and *ERF139* (ethylene-responsive transcription factors), *MEKK1* (MAP kinase kinase kinase), *PR-1* (pathogenesis-related protein 1), and *RBOHB* (respiratory burst oxidase homolog B). Conversely, genes involved in JA signaling, heat shock response, and pattern-recognition receptor signaling were downregulated, including *JAZ17*, *JAZ18*, *JAZ19*, and *JAZ20* (jasmonate ZIM-domain proteins), *HSP80*, *HSP83*, and *HSP90-1* (heat shock proteins), *CNGC1*, *CNGC14*, *CNGC16*, and *CNGC20*, *CDPK20*, nine LRR receptor-like protein kinases, *MAPKK1/2* and *MAPKK4/5* (MAP kinase kinases), five NB-ARC domain-containing resistance proteins, *RLK14*, *RBOHA* and *RBOHD*, *SGT1* (solanitine galacto transferase), and *WRKY22-like*.

At 2 dpi, the upregulated gene set expanded to include additional calcium sensors (*CDPK1-like*, *CDPK2*, *CDPK2-like*), more CNGC channels (*CNGC18*), additional JAZ proteins (*JAZ16*, *JAZ18*), LysM-containing receptor-like kinases (*CERK1*, *LYK2*), and WRKY transcription factors (*WRKY9*, *WRKY22*, *WRKY29*), alongside sustained expression of *ERF80*, *ERF87*, *ERF98*, *ERF139*, *MEKK1*, *PR-1*, and *RBOHB*. In contrast, downregulated genes included *CDPK1* and *CDPK20*, *CNGC1*, *CNGC14*, *CNGC15*, *CNGC16*, and *CNGC20*, *HSP80*, *HSP83*, *HSP90*, and *HSP90-1*, *JAZ17*, *JAZ19*, and *JAZ20*, eleven LRR receptor-like protein kinases, *MAPKK1/2* and *MAPKK4/5*, eight NB-ARC domain-containing resistance proteins, *RLK14*, *RBOHA* and *RBOHD*, and *SGT1*.

Notably, whereas *ERF87* and *RBOHB* maintained sustained upregulation at both time points, JAZ genes and LRR-RLKs exhibited progressive downregulation, suggesting a temporal shift from PAMP-triggered immunity toward effector-triggered immunity responses. Comparative analysis of the 1 and 2 dpi transcriptomes revealed that a subset of genes exhibited consistent expression patterns across both time points. Notably, ethylene-responsive transcription factors (*ERF80*, *ERF87*, *ERF98*, *ERF139*) and the MAP kinase kinase kinase *MEKK1* were continuously upregulated from 1 to 2 dpi, indicating sustained activation of ethylene signaling and MAPK cascades as central hubs in potato defense against *R. solanacearum*. This temporal persistence suggests that these genes function as master regulators coordinating downstream immune outputs, rather than merely transient stress responders.

### RNA-Seq validation and ERF gene expression profiling

**Fig. 4:**
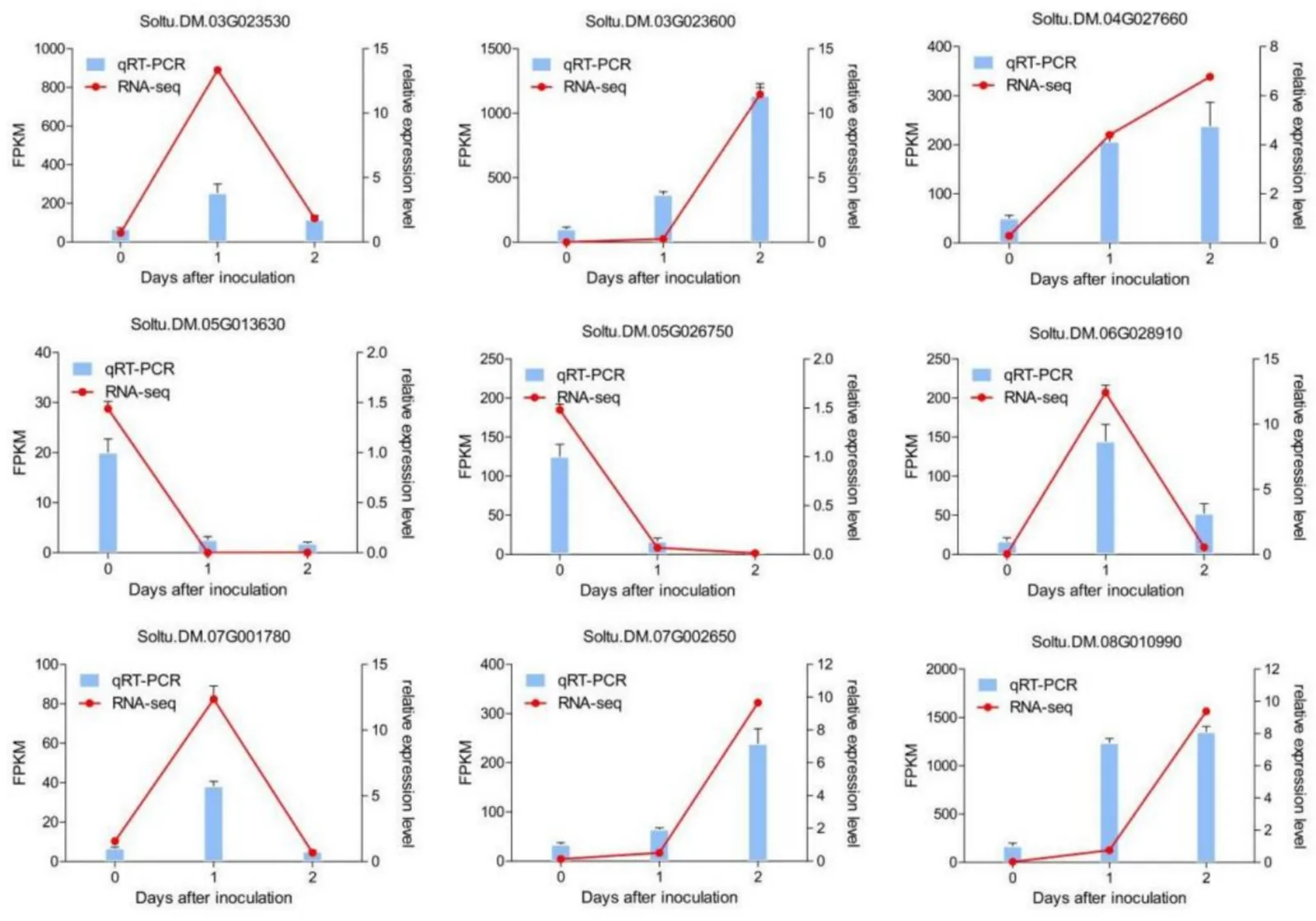
Validation of RNA-Seq data by qRT-PCR for 9 randomly selected DEGs. The x-axis indicates days post-inoculation (dpi) with *R. solanacearum*. The left y-axis shows FPKM values from RNA-Seq, and the right y-axis shows relative expression levels determined by qRT-PCR (2^−ΔΔCt^ method). Blue bars represent qRT-PCR data; red lines with dots represent RNA-Seq data. Data are presented as mean ± SD from three biological replicates (*n* = 3)

To validate the accuracy of transcriptome sequencing results, nine DEGs were randomly selected for qRT-PCR confirmation (Fig. 4), including *Soltu.DM.03G023530* (Kunitz family trypsin and protease inhibitor), *Soltu.DM.03G023600* (proteinase inhibitor type-2 CEVI57), *Soltu.DM.04G027660* (peroxidase), *Soltu.DM.05G013630* (phosphoglucomutase isoform X1), *Soltu.DM.05G026750* (chlorophyll a-b binding protein 6A), *Soltu.DM.06G028910* (cystatin B), *Soltu.DM.07G001780* (ribonuclease T2), *Soltu.DM.07G002650* (carboxypeptidase A inhibitor domain-containing protein), and *Soltu.DM.08G010990* (lipoxygenase).

 As shown in Fig. 4, the expression profiles determined by qRT-PCR closely mirrored those from RNA-Seq for the majority of tested genes. Both methods consistently captured the progressive downregulation of *Soltu.DM.05G013630* and *Soltu.DM.05G026750*, the transient induction of *Soltu.DM.03G023530*, *Soltu.DM.06G028910*, and *Soltu.DM.07G001780* peaking at 1 dpi, the sustained upregulation of *Soltu.DM.03G023600* and *Soltu.DM.07G002650*, and the continuous elevation of *Soltu.DM.04G027660* from 1 to 2 dpi. A minor discrepancy was observed only for *Soltu.DM.08G010990*: while RNA-Seq revealed a gradual upward trend, qRT-PCR detected a more pronounced increase with peak accumulation at 2 dpi, likely attributable to the higher sensitivity of qRT-PCR in resolving transient expression dynamics. Overall, the strong concordance between the two platforms confirms the reliability and accuracy of the transcriptomic dataset.

**Fig. 5:**
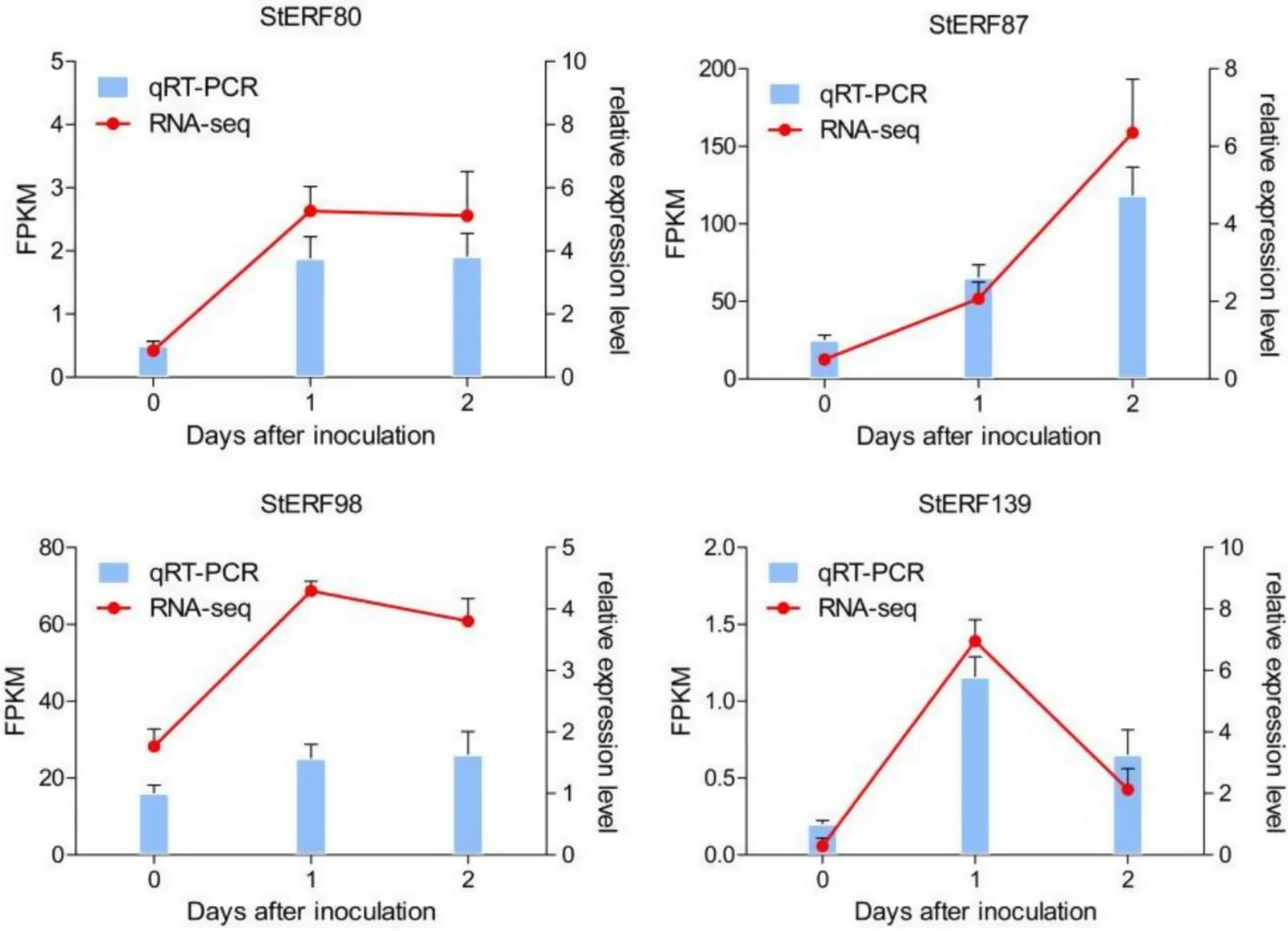
Expression patterns of differentially expressed ERF genes at different stages after *R. solanacearum* inoculation. The x-axis indicates days post-inoculation (dpi). The left y-axis shows FPKM values from RNA-Seq (red lines with dots), and the right y-axis shows relative expression levels from qRT-PCR (blue bars). Data are presented as mean ± SD from three biological replicates (*n* = 3)

Among the DEGs, members of the ERF family emerged as essential regulators of potato immunity against *R. solanacearum*. We therefore monitored the transcriptional dynamics of *ERF80*, *ERF87*, *ERF98*, and *ERF139* by qRT-PCR (Fig. 5). *StERF80* and *StERF98* exhibited approximately 3.7-fold and 1.6-fold increases in expression levels at 1 dpi, respectively; at 2 dpi, their expression levels remained unchanged compared to the first day. *StERF87* showed continuous induction of expression between 0 and 2 dpi, with peak levels reaching 4.7-fold. In contrast, *StERF139* reached a maximum induction level of about 5.8-fold at 1dpi, and then gradually decreased to around 3.5-fold at 2dpi. The results obtained from qRT-PCR were highly consistent with the trends observed in RNA-seq FPKM in the same figure, confirming the reliability of these data.

It is worth noting that although *StERF139* demonstrated the strongest relative induction effect, reaching up to 5.8-fold, its absolute expression level was still very low; the FPKM values were less than 2.0 throughout the testing period, indicating that its biological impact at the protein level is limited. *StERF87* exhibited the highest absolute expression level, with FPKM values exceeding 100 at 2 dpi; at 2 dpi, it showed a relatively high relative gene expression level, with an upward trend of approximately 4.7-fold. Similarly, *StERF98* maintained a relatively high baseline expression level, with FPKM values greater than 20; however, its response to induction was weaker, with only a 1.6-fold increase after infection with Rs. Therefore, *StERF87* was chosen as the subject of our study.

### *StERF87* overexpression confers enhanced resistance to bacterial wilt

**Fig. 6:**
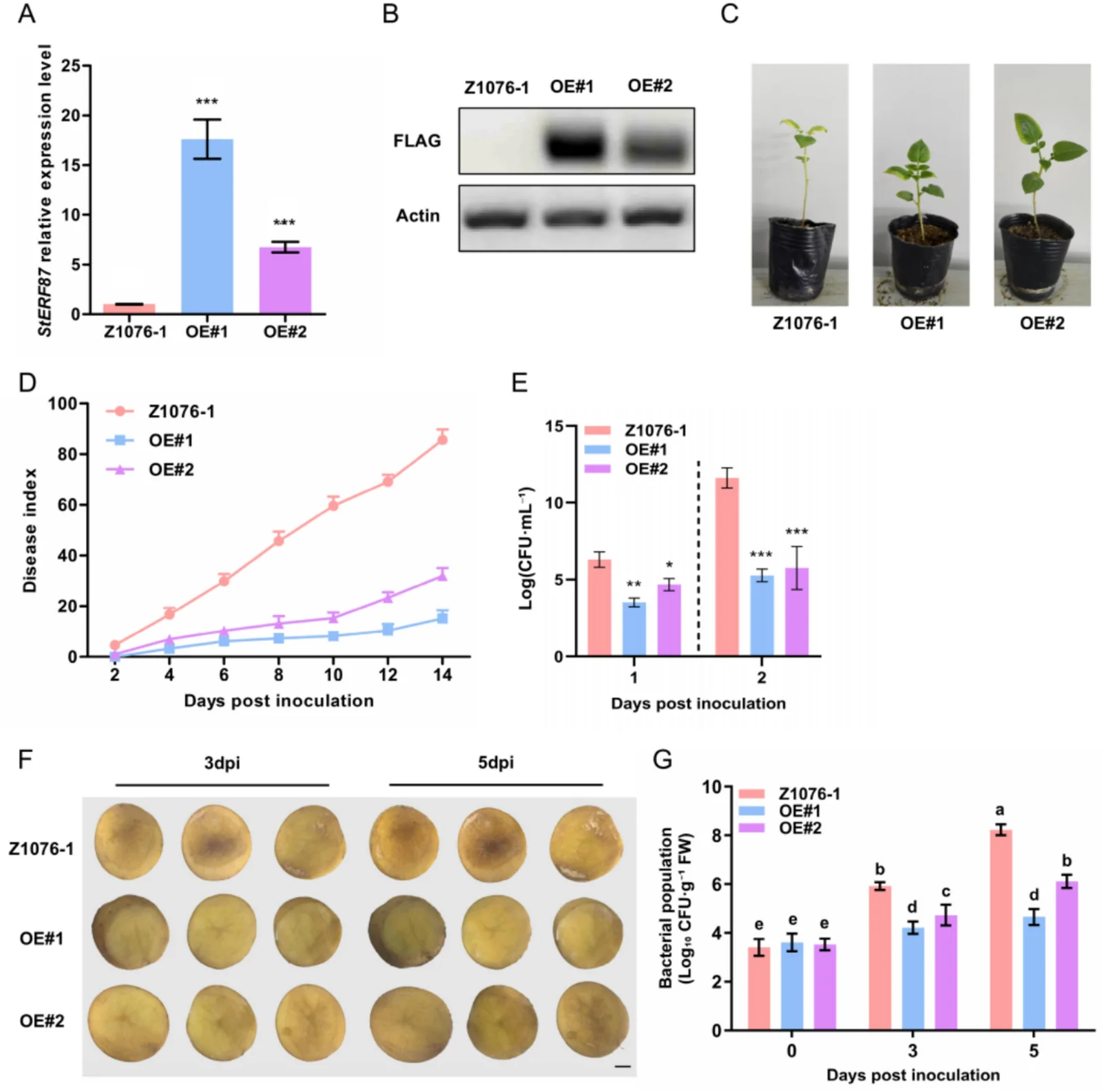
Enhanced resistance against *R. solanacearum* in *StERF87*-overexpressing transgenic plants. **A** qRT-PCR analysis of *StERF87* expression in two independent transgenic lines (OE#1 and OE#2) compared with wild-type (WT) Z1076-1. Expression in WT was normalized to 1. Data are means ± SD from three biological replicates. Asterisks indicate significant differences compared to WT (*P < 0.05, **P < 0.01, ***P < 0.001; Student’s t-test). **B** Western blot analysis of StERF87-FLAG protein accumulation in transgenic potato plants. Total proteins were extracted from leaves of Z1076-1, OE#1 and OE#2. The blot was probed with anti-FLAG antibody to detect the fusion protein and anti-Actin antibody as a loading control. **C** Representative phenotypes of *StERF87*-overexpressing and WT plants at 7 dpi. Four week-old plants were root-inoculated with *R. solanacearum* (10^6^ CFU mL⁻^1^) and maintained at 26 °C. **D** Phenotypic evaluation of disease resistance. Disease severity was scored on a 0–5 scale (see Materials and Methods). Data are presented as the mean Disease Severity Index (DSI) in percentage (%) with standard deviation from three biological replicates. **E** Bacterial populations in leaf extracts at 1 and 2 dpi, expressed as Log(CFU mL⁻^1^). Data are means ± SD (*n* = 3). Asterisks indicate significant differences compared to WT (*P < 0.05, **P < 0.01, ***P < 0.001; Student’s t-test). **F** Phenotypic observation of tuber slices from Z1076-1, OE#1 and OE#2 inoculated with *R. solanacearum* (10^6^ CFU mL⁻^1^) at 3 and 5 dpi. Scale bar = 1 cm. G, Bacterial population dynamics in tuber tissues. Data represent the mean ± SD (*n* = 5). Different lowercase letters indicate significant differences among different genotypes at the same time point (*P* < 0.05, Tukey’s test)

To elucidate the role of *StERF87* in potato defense, stable transgenic lines harboring the *35S:StERF87* construct were generated. PCR analysis revealed the presence of a specific band of approximately 700 bp in all 15 transgenic lines and positive controls. However, this band was not detected in Z1076-1 and negative controls (Fig. S3). Two transgenic lines with differing brightness of this band were selected for further experiments. qRT-PCR results showed that, compared to the wild-type Z1076-1, the levels of the *StERF87* transcript were significantly increased in the overexpressed lines OE#1 and OE#2; the level in OE#1 was approximately 17 times higher, while that in OE#2 was about 7 times higher (Fig. 6A). Further protein western blot analysis indicated that the StERF87-FLAG fusion protein was expressed normally in both OE#1 and OE#2, whereas no corresponding signal was observed in Z1076-1 (Fig. 6B). Four-week-old transgenic and WT plants were challenge-inoculated with *R. solanacearum*. Phenotypic evaluation at 7 dpi revealed that both OE#1 and OE#2 displayed substantially enhanced tolerance to the pathogen (Fig. 6C and Fig. S4). Disease severity indices were recorded from 2 to 14 dpi, showing that transgenic lines consistently exhibited lower disease severity than WT throughout the observation period (Fig. 6D). Furthermore, bacterial population quantification demonstrated that *R. solanacearum* titers in OE#1 and OE#2 were reduced compared to Z1076-1 at both 1 and 2 dpi (Fig. 6E). To assess resistance at the tuber level, tuber sections of Z1076-1, OE#1, and OE#2 were inoculated with *R. solanacearum*. At 3 dpi and 5 dpi, the tubers of OE#1 and OE#2 showed a significant increase in tolerance. Unlike Z1076-1, which exhibited obvious browning symptoms, OE#1 showed only mild symptoms, while OE#2 showed slight browning symptoms at 5 dpi (Fig. 6F). Quantitative analysis of bacteria in the tubers further revealed that at 3 dpi and 5 dpi, the load of *R. solanacearum* in OE#1 and OE#2 was significantly lower than that in Z1076-1 (Fig. 6G). It is worth noting that there was no significant difference in the bacterial load in OE#1 at 3 dpi and 5 dpi, indicating that the growth of the pathogen was effectively suppressed. These results establish that elevated StERF87 expression positively regulates potato defense against *R. solanacearum*.

**Fig. 7:**
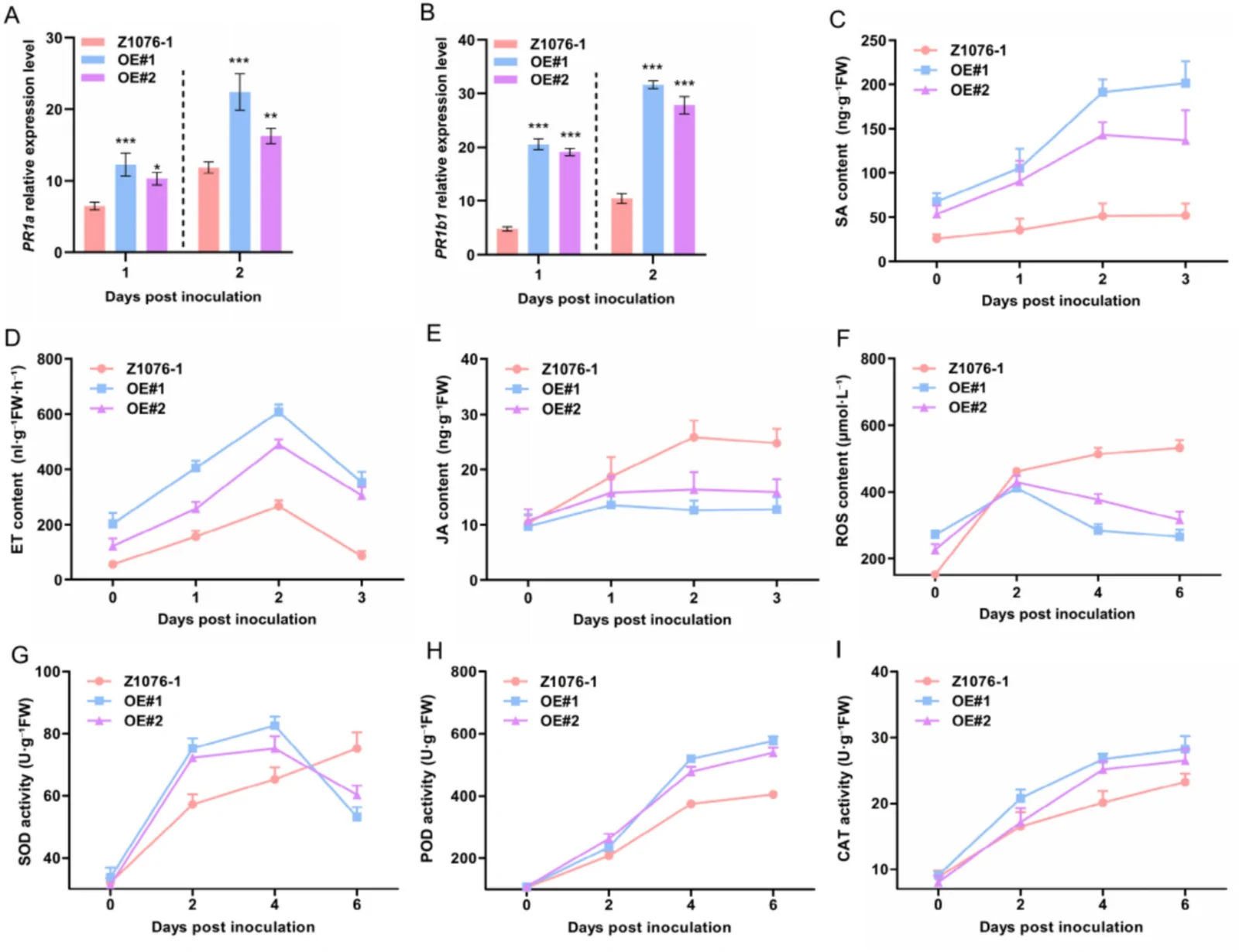
Defense-related gene expression, ROS content, and antioxidant enzyme activities in wild-type (WT) Z1076-1 and *StERF87*-overexpressing transgenic lines (OE#1 and OE#2) following *R. solanacearum* inoculation. **A–B** Relative expression levels of *PR1a* (**A**) and *PR1b1* (**B**) at 1 and 2 dpi. Data are means ± SD (*n* = 3). Asterisks indicate significant differences compared to WT (*P < 0.05, **P < 0.01, ***P < 0.001; Student’s t-test). **C–E** Dynamic changes in endogenous SA (C), ET (D), JA (E) levels at 0, 1, 2, and 3 dpi. **F–I** Dynamic changes in ROS content (**F**), SOD activity (**G**), POD activity (**H**), and CAT activity (**I**) at 0, 2, 4, and 6 dpi. Red, blue, and purple symbols represent WT, OE#1, and OE#2, respectively. Data are means ± SD (*n* = 3)

To investigate the molecular basis of StERF87-mediated resistance, the expression of immunity-related genes was monitored in Z1076-1 and StERF87-overexpressing lines following Ralstonia solanacearum challenge. *PR1a* and *PR1b1* were expressed at significantly higher levels in transgenic plants than in WT at both 1 and 2 dpi (Fig. 7A, B), suggesting that *StERF87* activates pathogenesis-related gene expression.

Corresponding hormone measurements revealed distinct profiles between genotypes. SA content was constitutively higher in OE lines at 0 dpi and continued to increase over time, reaching over 200 ng g^-1^ FW by 3 dpi, whereas the wild-type showed a more moderate elevation (Fig. 7C). ET emission rates followed a similar pattern: basal ET levels were elevated in transgenic lines, and a pronounced peak occurred at 2 dpi, with OE#1 reaching approximately 620 nl g^-1^ FW h^-1^—more than double the wild-type peak (Fig. 7D). In contrast, JA content increased in Z1076-1 after inoculation but remained at relatively lower levels in the transgenic lines throughout the time course (Fig. 7E).

Given that SA and ET accumulation are often accompanied by reactive oxygen species (ROS) production, we measured total ROS content and the activities of three major antioxidant enzymes. Total ROS increased in all genotypes after inoculation, peaking at 2 dpi (Fig. 7F). The transgenic lines exhibited a lower ROS peak compared to Z1076-1. SOD activity increased progressively in WT over the 6 day period, whereas in OE lines it peaked at 4 dpi and subsequently declined at 6 dpi (Fig. 7G). In contrast, POD and CAT activities were consistently higher in OE#1 and OE#2 than in WT at 4 and 6 dpi (Fig. 7H, I), although all lines showed sustained elevation of these enzymes throughout the observation period. Collectively, these findings demonstrate that *StERF87* overexpression enhances defense gene transcription and activates the antioxidant machinery, consistent with the enhanced tolerance observed in transgenic potato lines.

To determine whether StERF87 possesses intrinsic transcriptional activation activity, the full-length *StERF87* ORF was fused to the GAL4 DNA-binding domain (BD) in the pGBKT7 vector and transformed into yeast Y2HGold cells. The transformants harboring pGBKT7-StERF87 grew robustly on SD/-Trp/-His/-Ade/X-α-Gal (QDO) medium and exhibited strong blue coloration, similar to the positive control pGBKT7-p53, whereas the negative control pGBKT7-Lam did not grow on QDO medium (Fig. S5). These results indicate that StERF87 contains an intrinsic transcriptional activation domain and functions as a transcriptional activator.

### StERF87 directly binds to and activates the *StPR1a* promoter

**Fig. 8:**
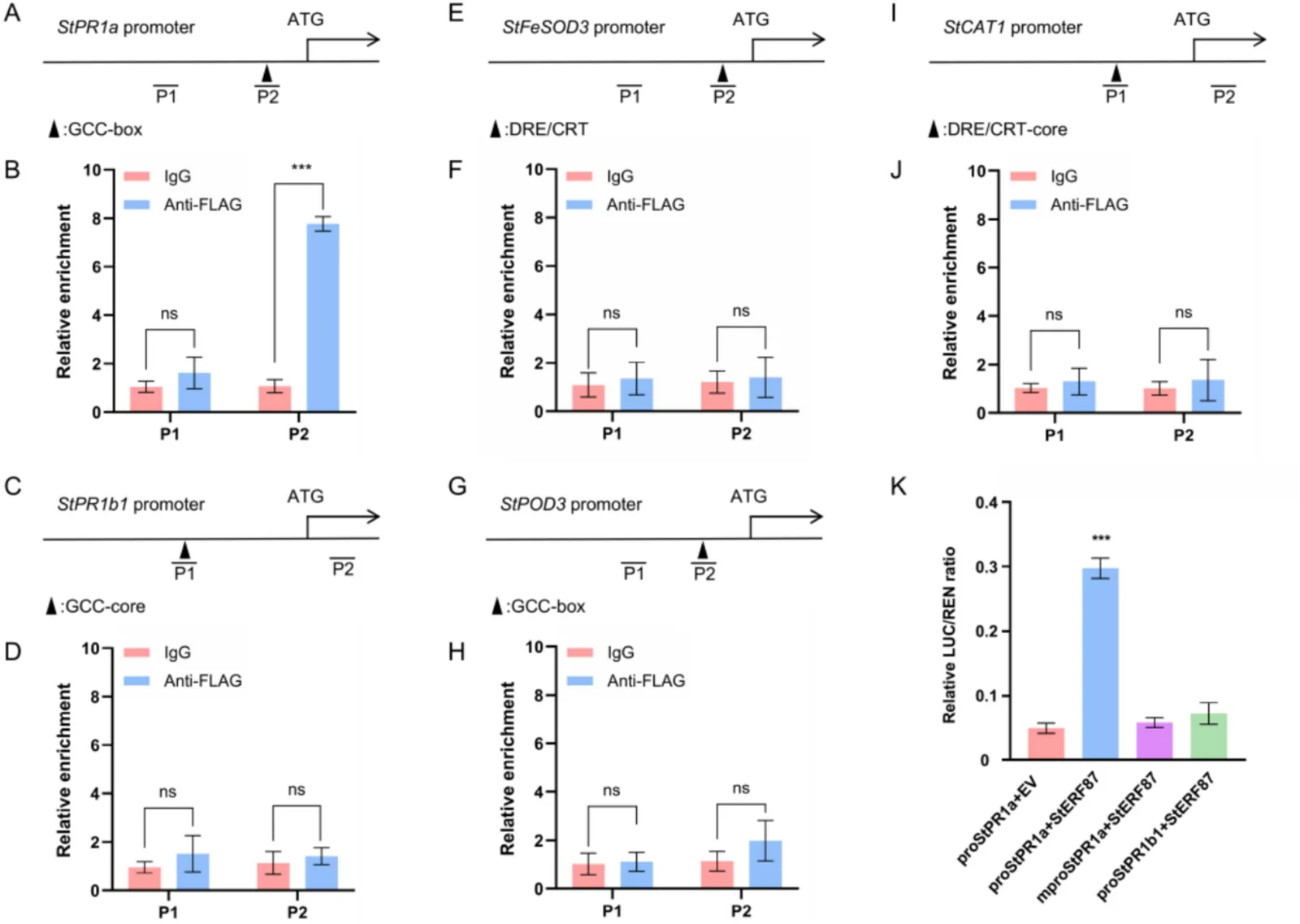
*StPR1a* acts as downstream genes of StERF87. **A** Schematic diagram of the *StPR1a* promoter regions. The black triangle represents the GCC-box motif. **B** ChIP-qPCR analysis of StERF87 binding to the promoter of *StPR1a*. **C** Schematic diagram of the *StPR1b1* promoter regions. The black triangle represents the GCC-core motif. **D** ChIP-qPCR analysis of StERF87 binding to the promoter of *StPR1b1*. **E** Schematic diagram of the *StFeSOD3* promoter regions. The black triangle represents the DRE/CRT motif. **F** ChIP-qPCR analysis of StERF87 binding to the promoter of *StFeSOD3*. **G** Schematic diagram of the *StPOD3* promoter regions. The black triangle represents the GCC-box motif. **H** ChIP-qPCR analysis of StERF87 binding to the promoter of *StPOD3*. **I** Schematic diagram of the *StCAT1* promoter regions. The black triangle represents the DRE/CRT-core motif. **J** ChIP-qPCR analysis of StERF87 binding to the promoter of *StCAT1*. **K** Dual-luciferase assay showing that StERF87 activates the *StPR1a* promoter. *mproStPR1a* designates the *StPR1a* promoter harboring a mutation (from AGCCGCC to AAAAAAA). Error bars indicate standard deviation, *n* = 5. Asterisks indicate significant differences compared to WT (*P < 0.05, **P < 0.01, ***P < 0.001; Student’s t-test)

To test whether StERF87 directly regulates *PR1* and antioxidant enzyme genes, we performed chromatin immunoprecipitation followed by quantitative PCR (ChIP-qPCR) using transgenic-expression material expressing 35S::FLAG-*StERF87*. We designed primer pairs flanking putative cis-acting elements in the promoters of *StPR1a* (GCC-box), *StPR1b1* (GCC-core), *StFeSOD3* (DRE/CRT), *StPOD3* (GCC-box), and *StCAT1* (DRE/CRT-core) (Fig. 8A, C, E, G, I). ChIP-qPCR results revealed a significant enrichment of StERF87 at the P2 region of the *StPR1a* promoter (Fig. 8B), where a GCC-box is located 300 bp upstream of the ATG start codon. No significant enrichment was observed at the P1 fragment of *StPR1a* or at either P1/P2 fragment of the *StPR1b1* promoter (Fig. 8D), indicating that StERF87 recognition is specific to the *StPR1a* promoter. Furthermore, StERF87 showed no significant occupancy at the promoters of the antioxidant genes *StFeSOD3*, *StPOD3*, or *StCAT1* (Fig. 8 F, H, J), implying that regulation of these genes by StERF87 is likely indirect.

To corroborate the ChIP-qPCR findings, we carried out a transient dual-luciferase reporter assay. Co-expression of StERF87 with the *StPR1a* promoter-driven reporter (proStPR1a:LUC) resulted in a strong and significant increase in relative luciferase activity compared with the empty vector (EV) control (Fig. 8K). In addition, dual-luciferase assays confirmed that StERF87 can significantly activate the promoter activity of the *StPR1a* gene. This was verified by an increase in the ratio of Firefly Luciferase (LUC) to Renilla Luciferase (REN). Importantly, when the AGCCGCC motif in the promoter of the *StPR1a* gene was replaced with AAAAAAA, the inhibitory effect of StERF87 on the LUC/REN ratio was eliminated (Fig. 8K). These results indicate that the *StPR1a* gene is a downstream target of StERF87.

## Discussion

Bacterial wilt, caused by *R. solanacearum*, is a devastating soil-borne disease that seriously threatens potato production in tropical and subtropical regions worldwide (Mansfield et al. 2012). Due to its broad host range and complex pathogenic mechanisms, analyzing early host responses at the transcriptome level is important for identifying resistance-related genes. Temporal analysis showed that at 1 dpi, calcium signaling components, ethylene-responsive transcription factors, and respiratory burst oxidase homologs were rapidly upregulated, whereas JAZ proteins and LRR receptor-like kinases were downregulated. This matches the characteristics of early pattern-triggered immunity (PTI) (Boller and Felix 2009). At 2 dpi, *ERF87* and *RBOHB* maintained sustained high expression, and the expression patterns of *WRKY* and LysM receptor-like kinases changed, suggesting a shift from early basal defense to later hormone-mediated specific defense (Jones and Dangl 2006). The sustained activation of ethylene signaling and the MAPK cascade may serve as core regulatory nodes in potato resistance to bacterial wilt, which is consistent with functional reports on ERF and MAPK components in crops (Lai et al. 2012; Chen et al. 2021).

In this study, we identified several ERF family genes markedly upregulated during potato resistance to *R. solanacearum*. Transcriptome profiling by RNA-Seq revealed strong expression of *ERF80*, *ERF87*, *ERF98*, and *ERF139* at both 1 and 2 dpi, and qRT-PCR subsequently corroborated this pattern (Fig. 5). Among them, at 2 dpi, *StERF87* exhibits a high absolute expression level, with FPKM values exceeding 100; moreover, the relative expression level is induced by a factor of up to 4.7. The expression levels of *StERF87* in the leaves and tubers of the *StERF87-OE* potato line were significantly increased. As a result, the severity of the disease was reduced, and the amount of pathogens present was decreased (Fig. 6). Recent reports indicate that among the differentially expressed genes in disease-resistant potato varieties, the ERF family is one of the transcription factor families with the highest expression levels (Chen et al. 2025). This highlights the important role of the ERF family in resisting *R. solanacearum*. Recent studies on ERF transcription factors in potatoes have demonstrated that *StERF3*, *StERF94*, and *StPti5* all participate in the potato’s biological stress response. Through phylogenetic analysis, it was found that StERF87, StERF94, and StPti5 belong to the same main branch; whereas StERF3 belongs to a different branch (Figure S6). Interestingly, even though they belong to the same branch, *StERF94* and *StPti5* perform opposite functions. Overexpression *StERF9*4 in potato plants enhances its resistance to *Fusarium solani* infection (Charfeddine et al. 2019). As a pathogenic factor, *StPti5* negatively regulates the immune response of potatoes to Potato Y virus and *R. solanacearum* (Coll et al. 2024). *StERF3*, which belongs to the other branch and has characteristics of typical EAR inhibitory factors, can improve the tolerance of potatoes to plant pathogens when it is downregulated or silenced (Tian et al. 2015; Razzaq et al. 2022). In terms of function, *StERF87* is more similar to the positive regulatory factor *StERF94*, rather than the negative regulatory factors *StPti5* and *StERF3*. This indicates that there are complex functional differences within the potato ERF family, and genetic relatedness alone cannot accurately predict their immunomodulatory effects.

Hormones help plants manage normal development and external stress (Lubyanova et al. 2021). When attacked by pathogens, salicylic acid (SA) helps the host respond by turning on defense mechanisms (Coelho et al. 2019). Systemic acquired resistance (SAR) works as a broad defense response. Signal molecules trigger this response after local infection by pathogens, including fungi, bacteria, and viruses. In SAR, SA acts as the "switch molecule". After pathogen entry, SA accumulates to high levels. This buildup then activates *PR* genes, among them *PR1* together with *PR2* and *PR5*, leading to systemic disease resistance (Kumar et al. 2025). This study found that, compared with the wild type, the OE lines maintained a higher basal SA level at 0 dpi and exhibited a continuous accumulation trend, with the content exceeding 200 ng g^-1^ FW by 3 dpi; the wild-type only showed a relatively moderate upward trend (Fig. 7C). The above results suggest that *StERF87* may enhance pathogen-induced SAR by regulating the synthesis or signal transduction of SA. Additionally, some studies have shown that plant hormones can act alone or work together through interactions between signaling pathways. The composition of specific hormones also affects plant disease resistance (Derksen et al. 2013; Amaro et al. 2023). This study found that in *StERF87*-overexpressing lines, both SA and ET levels increased, whereas JA levels remained lower (Fig. 7C–E). The elevation of SA and ET in OE lines likely counteracts the virulence strategy of *R. solanacearum*, which deploys type III effectors to suppress both SA biosynthesis and ET signaling. For instance, the effector RipAB directly targets *TGA* transcription factors to reduce SA-dependent immune gene expression and ROS production (Qi et al. 2022), whereas RipP1 suppresses the ethylene pathway through its interaction with the host protein FRL4a (Xie et al. 2026). The constitutive activation of these pathways in transgenic plants likely counteracts such pathogen-mediated immune suppression. The accompanying reduction in JA content reflects the well-documented SA–JA antagonism, a hormonal crosstalk extensively manipulated by *R. solanacearum* effectors such as RipAL, which induces JA production to actively suppress SA-mediated defenses (Nakano and Mukaihara 2018), and RipAF1, which ADP-ribosylates host FBN1 to mediate antagonistic crosstalk between JA and SA signaling (Wu et al. 2024). Collectively, these findings suggest that *StERF87* overexpression reprograms the hormonal balance toward an SA/ET-dominant defense program. *R. solanacearum* is known to deploy effectors that suppress both SA and ET signaling to facilitate its colonization of the vascular system (Chachar et al. 2025). Beyond hormone signaling, structural properties of the xylem may also contribute to resistance against vascular pathogens. Expression of fungal elicitors in banana xylem elevated JA levels and thickened xylem walls, enhancing resistance to Fusarium wilt (Negi et al. 2025). By contrast, JA accumulation in *StERF87*-overexpressing lines was markedly weaker than in wild-type plants following *R. solanacearum* inoculation. Xylem structural traits were not examined in this study, yet they represent an important SAR-related parameter for evaluating resistance to vascular pathogens. Future work should incorporate xylem anatomy to reach a more comprehensive conclusion on the resistance phenotype.

Pathogenesis-related (PR) proteins are key components of plant defense responses, with PR1 serving as a canonical marker of SAR (van Loon et al. 2006). In tomato, PR-1a1 has been reported as one of the most strongly upregulated genes following *R. solanacearum* infection (Li et al. 2025), supporting its relevance in bacterial wilt defense. In this study, we showed that StERF87 binds directly to a GCC-box motif in the *StPR1a* promoter and activates its transcription (Fig. 8B). The GCC-box (AGCCGCC) is a known binding site for the AP2/ERF domain of ethylene-response factors and is frequently present in the promoters of ethylene-inducible PR genes (Wu et al. 2022). This result indicates that StERF87 directly regulates *StPR1a* expression at the transcriptional level. A similar mechanism has been described in soybean; GmERF113 has also been shown to bind the GCC-box (Zhao et al. 2017). While StERF87 directly binds the *StPR1a* promoter, chromatin immunoprecipitation assays detected no binding to the *StPR1b1* promoter (Fig. 8D). This indicates that the up-regulation of *StPR1b1* observed in overexpression lines is not directly mediated by StERF87. SA treatment increases *PR1b* expression and ET signaling also activates basic PR-1 forms like *PR1b1* when pathogens infect plants (Sessa et al. 1995; Tornero et al. 1997). But JA does not induce *PR-1b* mRNA in potato (Hoegen et al. 2002). Because StERF87 overexpression increases SA and ET levels, the higher *StPR1b1* expression in transgenic lines may result from these hormone changes, rather than from direct regulation by StERF87. Taken together, our data demonstrate that StERF87 directly activates *StPR1a* via promoter binding, providing a mechanistic link between StERF87 and the transcriptional induction of this SAR marker gene in potato.

Plants rapidly produce a reactive oxygen species (ROS) burst after they recognize pathogens. This is an early hallmark of immune responses (Torres et al. 2006). ROS can directly inhibit pathogen growth and help strengthen cell walls. However, too much ROS accumulation causes lipid peroxidation and cell damage. Therefore, plants need to carefully control ROS balance through antioxidant enzyme systems (Mittler 2002, 2017). In this study, wild-type plants showed continuously increased ROS levels after *R. solanacearum* inoculation. This is consistent with previous reports that *R. solanacearum* infection creates an oxidative environment in tomato (Flores-Cruz and Allen 2009). In contrast, *StERF87*-overexpressing lines showed a rapid ROS increase within 2 days after inoculation. Then ROS levels gradually decreased and almost returned to the initial levels by day 6. This dynamic pattern suggests that *StERF87* may improve ROS scavenging ability. This helps plants remove excess ROS in time after defense signaling is completed. Regarding antioxidant enzyme activities, POD and CAT activities continuously increased after *R. solanacearum* inoculation. The changes were significantly greater in overexpression lines than in wild-type plants, and these activities kept rising at 4–6 dpi. In contrast, wild-type SOD activity kept increasing, but overexpression lines showed a decrease at 4–6 dpi. ChIP-qPCR results showed that StERF87 did not directly bind to the promoters of *StPOD3*, *StFeSOD3*, and *StCAT1*. This suggests that the changes in these enzyme activities were not caused by direct transcriptional activation by StERF87, but were achieved through indirect pathways. POD is involved not only in H₂O₂ decomposition, but also plays an important role in cell wall lignification (Almagro et al. 2009). In a study on sweet potato resistance to nematodes, resistant varieties still showed significantly higher POD activity than controls at 28 dpi, and CAT activity kept increasing in the late stage of infection (Yang et al. 2023). In comparison, SOD serves as the first line of defense in the antioxidant system, converting O₂⁻ into H₂O₂ (Apel and Hirt 2004). After *R. solanacearum* infection in tomato, antioxidant enzyme activities showed time-dependent differences, and the changing trends of different enzymes were not fully synchronized (Mandal et al. 2011). In this study, the decrease in SOD activity in overexpression lines at 4–6 dpi, while CAT and POD kept increasing, may reflect coordinated adjustment within the antioxidant system. After O₂⁻ was rapidly converted in the early stage, downstream CAT and POD enhanced their activities to remove accumulated H₂O₂. At this point, the need for upstream SOD decreased accordingly. This helps avoid oxidative damage and maintain defense signals at appropriate levels (Mittler 2017).

**Fig.9:**
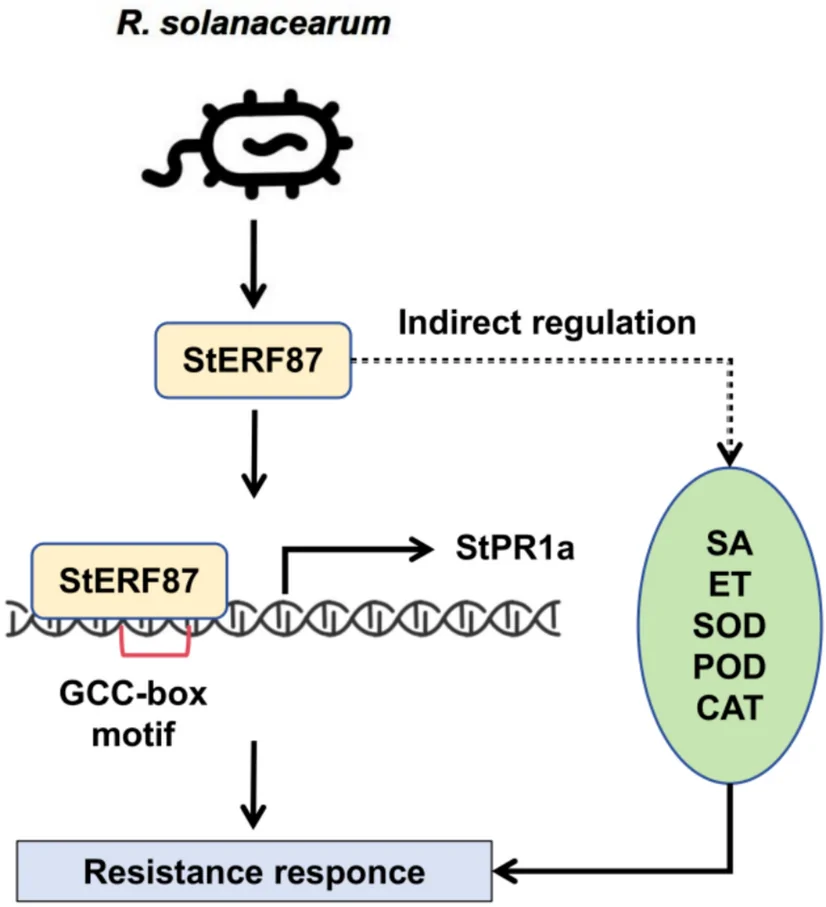
Working model of *StERF87*-mediated defense against *R. solanacearum* in potato. Upon pathogen infection, StERF87 is upregulated and binds the GCC-box in the *StPR1a* promoter to directly activate its transcription. Concomitantly, StERF87 elevates salicylic acid (SA) and ethylene (ET) levels and modulates ROS-scavenging enzyme activities (SOD, POD, and CAT), thereby protecting host cells from oxidative damage. The integrated output of these pathways—PR gene activation, hormone reprogramming, and ROS homeostasis—culminates in enhanced bacterial wilt resistance

Taken together, these findings allow us to propose a working model for the function of StERF87 in potato resistance to *R. solanacearum* (Fig. 9). In this model, upon infection by *R. solanacearum*, StERF87 controls defense through two pathways. First, it directly binds to the GCC-box in the *StPR1a* promoter and activates its transcription, which triggers SAR-related immune responses. Second, it indirectly affects hormone levels and antioxidant enzymes. It raises SA and ET levels and lowers JA levels. It also coordinates antioxidant enzyme activities: SOD activity is higher in OE lines than in WT from 1 to 4 dpi, while POD and CAT activities remain high from 4 to 6 dpi. At 6 dpi, SOD activity in OE lines falls below that of WT, which likely reflects a coordinated adjustment to avoid excess ROS after the early defense response. This dual ability to directly activate defense genes and indirectly regulate hormones and ROS balance makes StERF87 a promising candidate for breeding bacterial wilt resistance. However, since current data come from controlled conditions, multi-year and multi-location field trials are needed to fully test its breeding value. Furthermore, ChIP-seq and yeast two-hybrid studies are needed to find more target genes and interacting proteins. At the same time, the upstream signals that activate *StERF87* remain unclear. In banana, overexpression of *MusaNAC29-like* induces the expression of ethylene-responsive transcription factors and alters phytohormone content (Negi et al. 2023), suggesting that NAC transcription factors may act at a higher hierarchical level to regulate ERF-mediated defense. Whether StERF87 is subject to similar upstream control warrants further investigation. These will help us fully understand how StERF87 works and support its use in potato improvement.

## Conclusion

In conclusion, we identified *StERF87* as a positive regulator of potato defense against *Ralstonia solanacearum*. RNA-Seq identified 6663 and 7390 differentially expressed genes at 1 and 2 dpi. *StERF87* showed the highest absolute expression level among ERF members, with FPKM exceeding 100 at 2 dpi. Overexpression lines showed reduced disease severity and lower bacterial titers. StERF87 activated *PR1a* and *PR1b1* expression, increased salicylic acid and ethylene levels, and enhanced antioxidant enzyme activities. ChIP-qPCR and dual-luciferase assays confirmed that StERF87 directly binds the GCC-box in the *StPR1a* promoter to activate its transcription. These findings reveal the role of StERF87 in regulating potato defense and provide a basis for developing bacterial wilt-resistant varieties.

## Supplementary Information

Below is the link to the electronic supplementary material.

Supplementary file1 (DOCX 1913 KB)

Supplementary file2 (XLSX 15 KB)

Supplementary file3 (XLSX 789 KB)

Supplementary file4 (XLSX 890 KB)

Supplementary file5 (XLSX 12 KB)

## Data Availability

All transcriptomic, metabolomic, and physiological data generated during this study are available from the corresponding author upon reasonable request.
