# Supplementary material for: Transcriptomics and overexpression analyses reveal StERF87 confers resistance to Ralstonia solanacearum in potato
Source: Plant Cell Rep. 2026 Sep 8;45(10):284. doi: 10.1007/s00299-026-03967-7 (PMC13553665; doi:10.1007/s00299-026-03967-7)
Supplement: Supplementary file 1 [file 299_2026_3967_MOESM1_ESM.docx]

**Transcriptomics and Overexpression Analyses Reveal *StERF87* Confers Resistance to *Ralstonia solanacearum* in Potato**

Ru Yu^1*^, Min Gao^2^, Lin Cai^2^, Shuoxuan Wang^2^, Qingshuai Chen^1^, Peiyan Guan^3^, Caicai Lin^1^, Xia Zhang^1^, Shuangshuang Zheng^1^, Lingling Jiang^1^, Jihua Wang^1^, Linshuang Hu^1*^

^1^ Institute of Biophysics, Dezhou University, Dezhou 253023, China

^2^ Belgorod Institute of Food Sciences, Dezhou University, Dezhou 253023, China

^3^ College of Life Science, Dezhou University, Dezhou 253023, China

^*^ Correspondence: Yuru@dzu.edu.cn (RY); hulinshuang@dzu.edu.cn (LH)

ORCID: 0000-0002-5015-7087 (RY)

**Supplementary Table S1** Summary of RNA-Seq data and sequence assembly.

| **Sample** | **Raw reads** | **Clean reads** | **Raw bases (G)** | **Q20 (%)** | **Q30 (%)** | **Total**  **clean reads** | **Total mapped**  **reads (%)** | **Multiple**  **mapped (%)** | **Uniquely**  **mapped (%)** |
| --- | --- | --- | --- | --- | --- | --- | --- | --- | --- |
| Stc1 | 87605654 | 86795846 | 13.14 | 97.41 | 92.78 | 86795846 | 76.86 | 3.21 | 73.65 |
| Stc2 | 103105470 | 101744382 | 15.47 | 97.82 | 93.69 | 101744382 | 78.39 | 3.27 | 75.12 |
| Stc3 | 89963500 | 88136608 | 13.49 | 97.56 | 93.1 | 88136608 | 78.33 | 3.09 | 75.25 |
| St1Rs1 | 81774274 | 80596276 | 12.27 | 97.73 | 93.52 | 80596276 | 76.37 | 2.92 | 73.46 |
| St1Rs2 | 86078208 | 84739712 | 12.91 | 97.74 | 93.49 | 84739712 | 74.68 | 3.2 | 71.49 |
| St1Rs3 | 89186154 | 88052804 | 13.38 | 97.59 | 93.23 | 88052804 | 75.76 | 3.27 | 72.49 |
| St2Rs1 | 79959760 | 79157896 | 11.99 | 97.73 | 93.47 | 79157896 | 76.15 | 2.77 | 73.38 |
| St2Rs2 | 89637670 | 88597282 | 13.45 | 97.71 | 93.43 | 88597282 | 75.29 | 2.49 | 72.8 |
| St2Rs3 | 93893960 | 92554302 | 14.08 | 97.66 | 93.32 | 92554302 | 78.02 | 2.72 | 75.3 |

**Fig. S1** In vitro regeneration of potato plantlets from stem segments. A, Initial culture of stem segments. B, Shoot regeneration from stem segments. C, Plantlets with developed root systems. Scale bars = 1 cm.

**Fig. S2** Distribution of mRNA gene expression levels and clustering analysis of differential gene levels. A, Distribution of mRNA gene expression levels in each sample. The abscissa is the name of the sample (group), the ordinate is log10 ^(FPKM+1)^, and the box diagram of each region corresponds to five statistics (from top to bottom, respectively: maximum, upper quartile, median, lower quartile, and minimum value). B，Hierarchical clustering heatmap of mRNA differential genes. The x-axis represents samples, and the y-axis represents differential genes. The left panel clusters genes based on expression similarity, while the top panel clusters samples according to expression profile similarity, with expression levels increasing progressively from blue to red. Numerals indicate normalized relative expression values. St1RS1, St1RS2, and St1RS3 denote samples collected 1 day post-Rs infection, St2RS1, St2RS2, and St2RS3 denote samples collected 2 days post-Rs infection, and Stc1, Stc2, and Stc3 denote samples collected prior to Rs infection.

**Fig.S3** PCR identification of *StERF87* transgenic plants. M: DNA marker (2000bp); 1–15: putative transgenic lines; WT: wild-type plant (Z1076-1); NC: negative control (using deionized water as template); PC: positive control (using plasmid containing *35S::StERF87* gene as template). Lanes 5 and 6 (designated as OE#1 and OE#2, respectively) were selected for subsequent experiments.

**Fig. S4** Overexpression of *StERF87* enhances resistance to bacterial wilt in potato. Representative phenotypes of wild-type (WT) Z1076-1 and *StERF87*-overexpression transgenic lines OE#1 and OE#2 at 7 days post-inoculation (dpi) with *Ralstonia solanacearum*. Five independent biological replicates per line are shown.

**Fig. S5** Transactivation activity analysis of StERF87 in yeast. The full-length ORF of *StERF87* was fused to the GAL4 DNA-binding domain (BD) in the pGBKT7 vector and transformed into yeast strain Y2HGold. Transformants were spotted onto SD/-Trp (DDO) and SD/-Trp/-His/-Ade/X-α-Gal (QDO) plates. pGBKT7-Lam and pGBKT7-p53 served as negative and positive controls, respectively. Blue coloration on QDO medium indicates β-galactosidase activity and transactivation activity.

**Fig. S6** Neighbor-joining phylogenetic tree of potato ERF family members. The tree was generated using the full-length protein sequences of potato ERFs. Genes of interest involved in biotic stress responses are specifically marked: StERF87 (red circle), StERF94 (green triangle), StPti5 (blue square), and StERF3 (purple diamond). Numbers at the nodes represent bootstrap values.
